# The success of pharmacogenomics in moving genetic association studies from bench to bedside: study design and implementation of precision medicine in the post-GWAS era

**DOI:** 10.1007/s00439-012-1221-z

**Published:** 2012-08-25

**Authors:** Marylyn D. Ritchie

**Affiliations:** Department of Biochemistry and Molecular Biology, The Huck Institutes of the Life Sciences, Center for Systems Genomics, Eberly College of Science, The Pennsylvania State University, 512 Wartik Laboratory, University Park, PA 16802 USA

## Abstract

Pharmacogenomics is emerging as a popular type of study for human genetics in recent years. This is primarily due to the many success stories and high potential for translation to clinical practice. In this review, the strengths and limitations of pharmacogenomics are discussed as well as the primary epidemiologic, clinical trial, and in vitro study designs implemented. A brief discussion of molecular and analytic approaches will be reviewed. Finally, several examples of bench-to-bedside clinical implementations of pharmacogenetic traits will be described. Pharmacogenomics continues to grow in popularity because of the important genetic associations identified that drive the possibility of precision medicine.

## Introduction

Personalized medicine, or more recently coined precision medicine (Khoury et al. 2012), has advanced as one of the predominant strategic initiatives and goals of the next decade for many pharmaceutical companies, biotech institutes, academic medical centers, and the National Institutes of Health. The primary goal of this type of initiative is to treat patients with the correct dose of the appropriate medication based on their individual demographic and genomic makeup (Khoury et al. 2012; Mirnezami et al. 2012; Garay and Gray 2012). Pharmacogenetics and pharmacogenomics have made the dreams of precision medicine a reality. Pharmacogenetics is the study of a single genetic variant with a drug response phenotype, such as treatment responders and non-responders (i.e. assessment of drug efficacy) or a serious adverse side effect (i.e. drug toxicity). As molecular technologies to assay the entire genome have developed and genome-wide association studies (GWAS) emerged, so did pharmacogenomics (surveying the entire genome for associations with drug response phenotypes). As with other genetic traits and diseases, it is hypothesized that variability in drug response is due to underlying individual variation in genetic architecture. This drug response can include efficacy, serious adverse events, toxicity, or variability in target or maintenance dose. In general, pharmacogenomic studies and analysis approaches for these types of studies are very similar to standard genetic epidemiology studies for complex diseases, however, there are some subtleties that should be considered and these will be described in this review.

Efforts in pharmacogenomics have been fruitful and as such, very satisfying to researchers for many reasons. When genetic or genomic associations are identified for a particular drug adverse event, such that an individual with a certain genotype has a significantly increased risk to develop such a reaction upon drug exposure, this result can immediately be useful to patients and physicians in a clinic; assuming of course that an alternative treatment is available. Similarly if the genotype of a patient can be useful to predict the proper dose of a medication, this association also has clinical utility whereby a physician can use this genotype information to guide their dosing. Associations such as these have the possibility to make an impact on human health much more rapidly than genomic associations with complex disease risk such as type II diabetes or Crohn’s disease. It is not to say that such associations are not of critical importance to progress in the field and future drug development, diagnostics, or prevention strategies. However, translating complex disease risk associations into clinical practice requires additional years of research.

Another reason that pharmacogenomics has become a significant research area in human genetics is that the effect size for many identified genetic associations for pharmacogenomic traits are much larger than those for common, complex diseases. Table 1 shows a selected number of genetic associations for complex diseases as well as pharmacogenomic traits extracted from the NHGRI GWAS catalog which captures most of the genome-wide associations identified in GWAS (Hindorff et al. 2009) (http://www.genome.gov/gwastudies/). If one compares the odds ratio for the selected pharmacogenomic traits (the first four examples in Table 1) to those of the complex disease traits (the last three examples in Table 1), most of the effect sizes for the drug response phenotypes are much stronger. This allowed these pharmacogenomic associations to be identified with a smaller sample size (tens to hundreds of individuals in pharmacogenomics, where complex trait studies often need thousands to tens of thousands of individuals). Of course, this also means that the confidence intervals on the odds ratio estimates will be larger and the estimates may be biased and imprecise (Hosmer 2000; Harrell et al. 2001), however, many of these associations have been replicated. So while the effect estimates may not be precise, they are predominantly larger than those for complex disease phenotypes (the last three examples in Table 1). This difference in effect size may be due to the known, large environmental factor that is the drug exposure—which is not always present or known in complex traits.

**Table 1:** Comparison of common, complex disease associations with pharmacogenomics (PGx)

Trait	Chr	Gene	OR (CI)	Sample size	*p* value	References
PGx trait						
Response to tamoxifen in breast cancer	10q22.3	*C10orf11*	4.51 (2.72–7.51)	240 cases	6 × 10^−8^	Kiyotani et al. (2012)
Response to statin treatment	12p12.1	*SLCO1B1*	4.5 (2.60–7.70)	85 cases, 90 controls	2 × 10^−9^	Link et al. (2008)
Response to hepatitis C treatment	20p13	*ITPA*	25 (11.11–50.0)	303 cases	2 × 10^−25^	Tanaka et al. (2011)
Nevirapine-induced rash	6p21.32	*HLA*-*DRB1*-*DQB1*	3.1 (2.30–4.20)	201 cases	5 × 10^−14^	Lucena et al. (2011)
Complex disease trait						
Type II diabetes	10q25.2	*TCF7L2*	1.46 (NR)	2,413 cases, 2,392 controls	2 × 10^−15^	Kho et al. (2012)
Obesity	16q12.2	*FTO*	1.39 (1.27–1.51)	685 obese children, 685 lean children	1 × 10^−28^	Meyre et al. (2009)
Age-related macular degeneration (AMD)	1q31.3	*CFH*	3.11 (2.76–3.51)	2,978 cases, 2,859 controls	2 × 10^−76^	Chen et al. (2010)

Associations from the NHGRI GWAS Catalog (Hindorff et al. 2009)

Finally, for some pharmacogenomics outcomes the study is relatively straight forward to design because the drug in question is well characterized and its mechanism of action is well known. This makes targeted genotyping or sequencing experiments feasible to design and implement. On the contrary, many drugs have an unknown mechanism of action and/or little is known about its metabolism or transport. This type of study lends itself to a dense, genome-wide assay (such as GWAS, exome sequencing, exome chips, or whole-genome sequencing). So prior knowledge about the drug can play a role as a strength or weakness for pharmacogenomic studies and it clearly plays an important role in the type of molecular approach selected for the study.

A limitation of pharmacogenomics research is the reality of study design constraints (Grady and Ritchie 2011). Because many pharmacologic agents exert great patient cost, both in terms of dollars as well as in disease treatment efficacy or risk of toxicity, it is not often the case that the study can be designed in an optimal way for genetic or genomic research. It is, for example, unethical to have a control group with disease who are denied drug treatment to compare to the disease group on drug. If the drug is FDA approved with proven patient benefits, it is not advisable to deny treatment to patients specifically to control the genomic study design. Therefore, it is more often the case that performing an exposed-versus-unexposed study is not possible. However, designs which included case-only on drug, with and without serious adverse events can be used. This is the most common design currently used (described more below). Another challenge related to study design is that most pharmacogenomic research studies are amended to existing projects. For example, many prospective clinical trials add a retrospective pharmacogenomics component. The limitation here is that pharmacogenomics researchers are confined to the original study design, which may or may not fulfill their research question.

Replication of effects is another significant challenge and limitation of pharmacogenomics studies, as compared to complex disease association studies. To replicate detected associations, one needs to have an independent study with the same drug treatment and phenotype outcome collected (adverse event, toxicity, etc.). For efficacy or dosing outcomes, one needs not only the same drug, but also the same dosing. For assessing drug–drug interaction associations, one needs the same drug cocktail observed in multiple patient cohorts. This presents a significant challenge. Often, because of this limitation, pharmacogenomics researchers focus on functional validation of associations in cell lines (Welsh et al. 2009; Duan et al. 2009; Huang et al. 2007; Matsson et al. 2012; Ingle et al. 2010) or model systems, rather than replication of effects.

Lastly, many pharmacogenomics traits or drug response studies have very limited sample size. Drugs that exhibit life-threatening adverse events, such as toxicity, are often pulled from the market. Even without intervention in this manner, many adverse events are quite rare. So while the effect size if often larger for pharmacogenomic traits, the available sample size may be appreciably smaller.

Regardless of the limitations described above, pharmacogenetic and pharmacogenomics studies have been extremely successful in human genetics. The ability to translate genetic associations from “bench to bedside”, which is the promise of translational research, has been demonstrated for several pharmaceuticals and various drug-related phenotypes (described in this review). It is clear that in this GWAS era, with thousands of known genetic associations for hundreds of traits, a type of study that has forged ahead, with great success is pharmacogenomics. In this review, the most common study designs for pharmacogenomics will be described. Next, the molecular and analytic strategies suited for pharmacogenomics will be briefly discussed. Finally, a number of the translational success stories of pharmacogenomics traits will be reviewed. This manuscript will provide evidence for the impetus in pharmacogenomics as an emerging area for human genetics.

## Epidemiologic study designs for pharmacogenomics

**Fig. 1:**
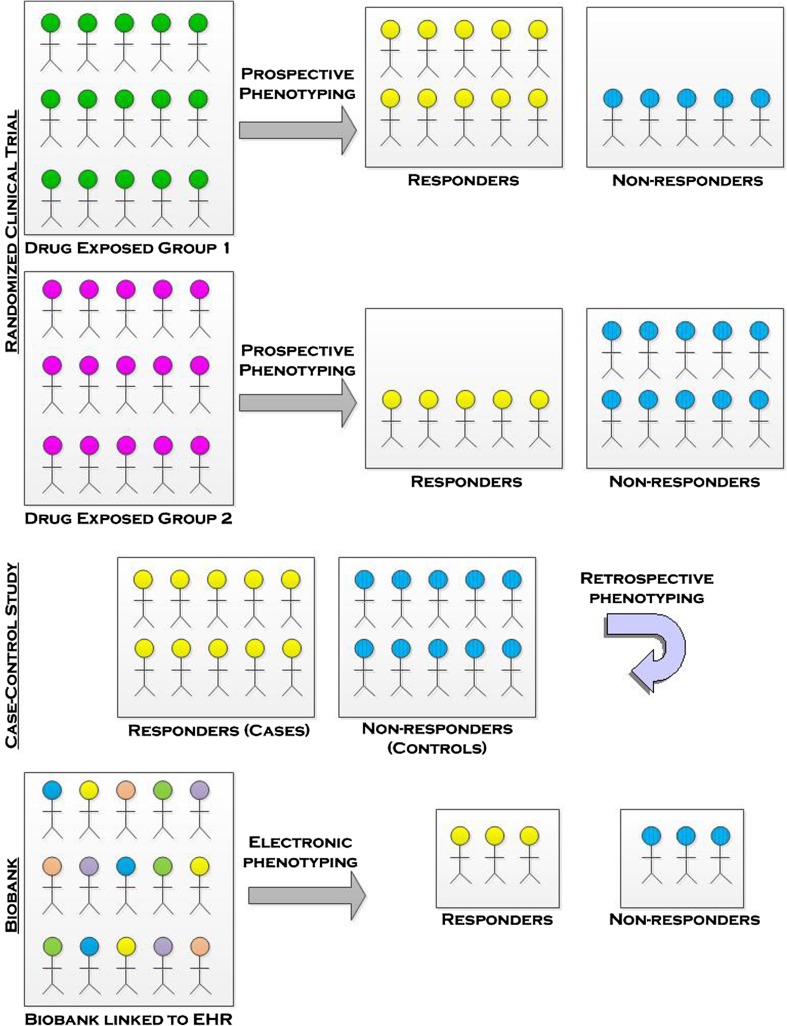
A visual display of the three primary epidemiologic study designs used in pharmacogenomics: randomized clinical trials, case–control, and biobanks

Pharmacogenomic studies are often performed on data collected for other pharmacologic research, although in some cases, prospective clinical trials have been designed specifically for pharmacogenomics testing. The three primary epidemiologic study designs used include clinical trials, retrospective case–control studies, or biobanks linked to electronic health records (EHR) as shown in Fig. 1. Each of these designs will be discussed briefly in the following sections, including strengths and limitations of each design. A summary of the strengths and limitations is presented in Table 2.

**Table 2:** Comparison of three study designs for pharmacogenomics

Randomized controlled trials	Observational case–control	Biobanks
Strengths		
Little confounding	Powerful analytic approach	Phenotypes can be selected after sample collection (from EHR)
Little selection bias	Control recruitment sample size of cases and controls	Many phenotypes are possible
Pristine phenotypes on short-term outcomes and toxicity	Can be prospective or retrospective	Patients are followed over time as they continue in clinic
Limitations		
Mid-trial changes due to toxicity can cause problems for research analysis	Bias from differential recall	Study design limited by what phenotypes/traits collected in the EHR
Population stratification	Population stratification	Population stratification
Potential for bias in DNA collection	Complications in phenotype collection (adherence, changes, multiple treatments)	Data collected for clinic purposes—not research
Cost: expensive in terms of time and money to follow participants	Cost: most data collected at study initiation; subsequent cost in making the data useful for analysis	Cost: bioinformatics for phenotyping is significant in terms of time and money

### Randomized clinical trials

A randomized clinical trial (RCT) is the gold standard of study designs in drug treatment-related research (Stolberg et al. 2004). An RCT is a well-designed study typically focused on understanding the efficacy and/or toxicity of a new therapeutic. Study participants are randomized to one of multiple treatment arms, which controls confounding and selection bias (Manolio et al. 2006). In addition, RCTs are usually conducted in a double-blind manner, where neither the study participants nor the researcher collecting the data is knowledgeable of the treatment arm to which the patient has been assigned (Stolberg et al. 2004). This process protects the study from bias in two ways. First, the study participants are less likely to have adherence issues, differential recall of symptoms, or placebo effects. This is due to the fact that participants are under the care of the study physician and coordinators and are typically seen in clinic at regular intervals, and they are all asked the same questions about their treatment effects. Second, this protects the research from evaluating participants in a biased manner (probing more for symptoms or minimizing severity of symptoms). The randomization and blinding processes attempt to control for heterogeneity and bias that could contribute to the results of the study and are major strengths of the RCT design.

Another significant strength of the RCT is the ability to collect pristine phenotype information (i.e. outcomes observed right after drug treatment and toxicities) as the patient population is being closely monitored during treatment initiation. In addition some RCTs have control arms which allow for the true assessment of the predictive ability of genetic markers to be realized, and to determine if the effect is pharmacogenomic or just genetic in nature. This provides more informative power than even the observational clinical trial designs which can only be prognostic. RCTs are otherwise epidemiologically sound experiments that allow one to observe prospectively the impact of therapeutics on the patient population. Finally, within an RCT, a case-only design can be used to look for gene–environment interactions where the “environment” is the drug (Little et al. 2005).

The strengths of the RCTs are clear whereas the limitations may or may not occur (depending on the trial design). For example, if there is a complication or toxicity due to treatment during the trial, regardless of which arm an individual was randomized to, their treatment may be modified during the study to accommodate their complications and/or changing symptoms. The safety and well-being of the participants outweighs controlling the proper study design. However, these issues can cause subsequent analyses of the trial data to be compromised. An additional limitation is that while randomization of study participants is attempted, it is not guaranteed. Therefore, researchers may still need to adjust for covariates that are not even distributed between groups. Another potential issue is that of biased DNA sample collection where not all participants are required to consent for DNA contribution, in addition to the reality that many RCT are multi-site studies and in some cases, not all sites collect biospecimens for DNA extraction or they collect samples in different ways (blood, saliva, etc.). As a result, systematic exclusion or bias of certain subgroups of participants may occur which could create significant issues with subsequent genetic association analyses. Finally, as with any large study followed for a period of time, there is significant expense related to maintaining the cohort. Not only is cost a potential issue, but also keeping track of participants over long periods of time can be complicated, if not impossible depending on the pharmacogenomic endpoint of interest. Thus, the dataset generated in the end of an RCT may be a substantially different sample size from what was predicted at the outset of the trial.

### Retrospective observational study

An alternative to the RCT which is more commonly used and certainly less expensive is a case–control study, or retrospective observational study. Case–control design has become the workhorse of genetic association studies in human genetics, as it has been shown to be the most powerful design for the detection of common variants associated with common traits (Kraft and Cox 2008). In this design, participants are enrolled based on their phenotype, or drug response (efficacy, adverse event, toxicity, etc.), and information regarding their exposures are collected retrospectively. This includes any number of exposures (medical, environmental, comorbidities, demographic, etc.) as well as a DNA sample for genotyping. A major strength of the case–control design, in addition to being a powerful approach, is the ability to control the recruitment sample size for the cases and controls. Typically, in pharmacogenomic studies, this design is used in a collection of individuals who are all receiving treatment for a particular disease. The “cases” and “controls” for the pharmacogenomic study are those affected and unaffected with some adverse event, toxicity, or the responders and non-responders (efficacious versus non-efficacious) to the treatment. Depending on the frequency of the side effect or toxicity, the potential sample size collected may vary quite a bit. Though less common, in some cases, a case–control study could be constructed from a prospective observational cohort study (also called a nested case–control study) such as in (Link et al. 2008). In this design, phenotype information is collected over time, and the case–control study is designed subsequently, depending on the outcomes observed and collected during the study. This type of study is most common in clinic-based biobanks (described in the following section). Case-only designs can also be extracted from case–control studies, and are often done to look specifically for gene–drug interactions (Little et al. 2005). An important consideration for the case-only design is the assumption that the gene and the environmental factor (drug) must not be correlated in the patient population. This assumption is essential for the G × E interaction to be valid.

A limitation of the case–control design is the potential bias introduced in any retrospective study in terms of differential recall. The nature of a retrospective design relies on participants to report their past exposures, symptoms, etc. (with the exception of the biobanks liked to electronic health records where the information comes from the medical record, not directly from the patient). This information is easier for some individuals to recall than others; which can introduce some degree of bias (Swan et al. 1992). For example, often “cases”, or in pharmacogenomics individuals who have toxic adverse events or individuals who do not respond to treatment (non-efficacious), on average these individuals may be more likely to recall their exposures including the drug, potential drug–drug interactions, other environmental or diet exposures, etc. because they think in depth about why their drug treatment did not work for them. Whereas in “controls”, or individuals where the drug showed expected efficacy or no toxic adverse event, they may not be as detailed in their recall of other drugs, environmental, or diet exposures because they have no need to. There are a number of epidemiological survey techniques used to control this issue, which can protect from these biases (Lash and Ahern 2012; Pathak et al. 2011; Stover et al. 2010; Hamilton et al. 2011; Pan et al. 2012; Hendershot et al. 2011).

Another limitation, which is also true of any retrospective clinical trial or biobank as well, is population stratification. As with any other population-based genetic association study, there is a risk of identifying allele frequency differences that are due to underlying population differences between cases and controls, rather than allele frequency differences due to disease (Balding 2006). Much work has been done in this area to identify evidence for population stratification and approaches to deal with it when it happens (Edwards and Gao 2012). In pharmacogenomics, an additional challenge exists when a particular serious adverse event or toxicity is more prevalent in one ancestral population. For example, Stevens Johnson syndrome (SJS) is associated with alleles in *HLA* and carbamazepine treatment and is more common in individuals who are of Asian descent (Locharernkul et al. 2011). To determine which alleles are associated with SJS, it is important to compare individuals without SJS to those with SJS from the same ancestry group. Otherwise any differences detected may be associated with ancestry group. This challenge is expanded even further when the research is considering individuals from admixed populations. If segments of the chromosomes originate from different ancestral populations, mapping the region harboring susceptibility loci can be particularly challenging. Most studies involving admixed populations will consider local ancestry and/or admixture mapping.

Another limitation of a retrospective design is a set of complications with study design that are typically out of the researcher’s control. These include combination of therapies, adherence to medication schedule, changes in dose or drug based on patient response. These needs to be dealt with after the data are collected and can lead to studies requiring stratified analyses or even dropping some individuals from the study. Lastly, the costs associated with a case–control design are primarily at the time of sample collection and enrollment, as all of the data are collected at one time point. The result is additional cost in extracting useful phenotype information from the data collected. The extraction of phenotype can vary in complexity, based on the depth of information collected. Despite the limitations, the case–control design is a very common approach for pharmacogenomics.

### DNA biobanks

The third study design which has been emerging in pharmacogenomics is a medical facility-based biobank linked to electronic health records (EHR) (or a similar cohort linked to medical records, White et al. 2011; Wilke et al. 2008; Kiyotani et al. 2008; Matimba et al. 2008). This is a type of prospective, observational study. The availability of electronic health records has been increasing dramatically in recent years (Kho et al. 2011). The ability to conduct genomic research from these records has been demonstrated in a number of recent studies (Denny et al. 2011; Crosslin et al. 2012; Kullo et al. 2011). The eMERGE network (electronic MEdical Records and GEnomics) which is funded by the National Human Genome Research Institute (NHGRI) of the National Institutes of Health in the United States, has driven this study design over the past several years (McCarty et al. 2011). Many health care providers, academic medical centers, and insurance providers have implemented DNA biobanks to link to their EHR systems for research purposes. From these cohorts of patient samples, pharmacogenomics studies can be developed. For example, an evaluation of warfarin dosing from samples extracted from the Vanderbilt biobank, BioVU, using genotypes from *VKORC1, CYP2C9* and *CYP4F2* has demonstrated the ability to extract pharmacogenomics phenotype information using electronic algorithms (Ramirez et al. 2012). In a study of tacrolimus response, *CYP3A5*/*CYP3A4* was identified previously to be associated with circulating drug levels and this effect was replicated in the Vanderbilt DNA biobank (BioVU) (Birdwell et al. 2012). Similar such studies can be designed and implemented when large patient populations have been collected with drug exposures and phenotype outcomes (such as adverse events or drug toxicity). This is a major strength of this design.

Like any cohort design, a number of genetic association studies can be constructed after the cohort has been established and populated. However, there are also limitations with this type of sample collection. The primary limitation is that the ability to perform any particular association study relies on the availability of samples with the particular phenotype or drug exposure of interest. Since there is little, if any, control exerted on the types of patients collected, it can be very difficult to have a large enough sample size to conduct a powerful association study. Similarly, in a biobank linked to an EHR, medical information is collected in the arena of patient care which may or may not have all of the necessary information for research purposes. Finally, phenotyping the patients to determine efficacy or drug toxicity requires significant medical informatics algorithm development. This can have varying levels of complexity depending on the trait, which then relates to the cost of phenotyping, which can be substantial for complex drug effects (such as neuropathy or liver toxicity). Thus, the pharmacogenomics design in a biobank linked to an EHR may be a powerful approach (as shown above) or it may be a challenge to accumulate sufficient sample sizes.

### In vitro study design

Many pharmacogenomics studies rely on epidemiologic designs described in the previous section, however, for many drugs this is not a practical approach. Variation in drug response may be due to multiple genes, each with small effects. In these cases, large sample sizes will be needed to identify the effects. This is unlikely to be the case in clinical trials, prospective cohorts, etc. Another complication is controlling potential confounding factors such as comorbidities, dosing, and diet. For all of these potential issues, many groups have relied on in vitro study designs using human cell-based models for pharmacogenomics discovery, as well as validation. In these experiments, cell lines are perturbed with drug treatment, in different doses, and changes in gene expression and cell growth can be observed (Welsh et al. 2009). The in vitro cell-based study design also has clear strengths and limitations, much like each epidemiologic study design. In terms of strengths, the experiment can be well controlled, large numbers can be generated in a cost-effective manner, and the samples are unlimited in terms of resources (as more cell lines can be made as needed) (Welsh et al. 2009). However, these studies are limited by the following: (1) must select cell lines from one tissue, and can only be made from certain tissues (may or may not be the right one for the drug in question, (2) cell lines may not express important enzymes needed for drug metabolism and transport, and (3) establishing cell lines may damage the cell in unknown ways, thus altering the cell’s characteristic and subsequently the pharmacogenomics response (Welsh et al. 2009). Still, cell lines, such as the HapMap lymphoblastoid cell lines (LCLs), have been used for several pharmacogenomics studies (Duan et al. 2009; Huang et al. 2007; Watson et al. 2011a, b; Brown et al. 2011), and have shown enormous potential.

## Molecular techniques for pharmacogenomics

Pharmacogenomics studies are inherently quite similar to standard human genetic studies; however, the phenotype is related to treatment response rather than simply a quantitative trait or disease risk. Due to the similarity, standard molecular technologies and analytic approaches can be applied to these studies (Grady and Ritchie 2011). For example, in the post-GWAS era, any number of data generation techniques can be used, depending on the scientific questions and hypothesis being tested. For example, if it is hypothesized that rare, coding variants will be most important for the pharmacogenomics trait of interest, exome sequencing or exome chips would be the most likely methodology of choice. Conversely, if gene expression variation from eQTLs (expression quantitative trait loci) or epigenetic variation are hypothesized to be most relevant, next generation sequencing of either RNA (RNAseq) or methylation sites (methyl-seq) may be selected instead. With the rapid development of novel technologies, there are a number of assays that can be considered and these have been reviewed elsewhere (Krueger et al. 2012; Zhou et al. 2011; Zhang et al. 2011; Ku et al. 2011). An important consideration is also the type of tissue being collected and the appropriate molecular technique selected. For example, when assaying germline DNA variation, DNA from blood (i.e. lymphocytes) would be appropriate and sufficient. However, if DNA variation of interest is related to somatic changes, such as in cancer, DNA from the tumor would be more appropriate. In addition, the comparison would typically be tumor tissue compared to a healthy section from the same tissue; this adds additional effort to perform such an experiment. If gene expression or epigenetic variation in the liver is of interest, surrogate tissue, such as blood, may be inappropriate for this assessment as blood cells may or may not reflect the actual relationship between drug and the organ affected by toxicity (i.e. liver, if metabolized by liver; skin if skin toxicity; etc.). A full survey of these techniques is out of the scope of this review, thus readers should be encouraged to explore some of these references (Manolopoulos et al. 2011; Kacevska et al. 2011; Midorikawa et al. 2012).

As mentioned earlier, an additional benefit to some pharmacogenomics studies is the knowledge of the mechanism of action of the drug. This can make the initial design of the molecular study much more targeted. For example, several companies have designed genotyping arrays specifically focused on drug metabolism and transport genes, such as the Affymetrix DMET chip and the Illumina ADME chip. Platforms like this will allow for more targeted evaluation of genes known to be related to the drug and/or phenotype of interest. Alternatively, a targeted exome or genome capture experiment could be considered if there is a list of genes hypothesized to be relevant for the drug metabolism. This approach will most likely only be relevant until the cost of whole-genome sequencing drops. Once the cost of sequencing the entire genome is low enough, this will be the method of choice as it allows one to obtain the rare variants as well as the common variants and everything in between. Still, even if the genes/pathways which control drug mechanism are known, these may or may not explain variation in response. So while this knowledge may guide the initial molecular assays, subsequent genomic assays may be needed. This will result in genome-wide genotyping or whole-genome sequencing being selected as the assay of choice.

## Analytic techniques for pharmacogenomics

Standard analysis techniques are typically implemented in pharmacogenomics studies. In general, the study design allows for standard regression methods, Chi-square tests, Cox-proportional hazard models for time to event analysis, or Wilcoxon tests, etc. and there is no need for specialized statistical methods. The only caveat to this is that the definition of case–control groups and the interpretation of results must be carefully considered. For example, if the case group is defined by a serious adverse side effect to statin treatment, and the control group is a population-based control group that includes a set of individuals who are not exposed to statins, associations identified may be more likely to be associated with the reason that the “cases” are prescribed statins, rather than the statin side effect. It is also possible that the association is important for the side effect. A mechanism to control for disease indication that led to treatment is needed for a study such as this.

Analysis techniques for pharmacogenomics have also been extensively reviewed (Motsinger et al. 2006a, 2007; Motsinger and Ritchie 2006; Flynn 2011; Rodin et al. 2011; Srinivasan et al. 2009). Briefly, for single SNP or variant analysis in pharmacogenomics association studies a large array of statistical methods can be utilized(Cantor et al. 2010) such as Chi-square test (Greenwood 1996; Zheng et al. 2004), Armitage trend test (Armitage 1955; Cree et al. 2010), Kaplan–Meier survival curves (Kaplan and Meier 1958; Huang et al. 2009), Bayesian statistics (Stephens and Balding 2009), or data mining methods (Coassin et al. 2010) but is commonly performed in the framework of regression (Woodahl et al. 2008). Most of these are performed in standard statistical analysis software such as STATA, SAS, R, PLINK, or others. Epistasis, or gene–gene interactions, and gene–environment interactions can be investigated through the use of many standard statistical methods (Motsinger et al. 2007; Cordell 2009), complex regression methods including lasso regression (Ayers and Cordell 2010) and logic regression (Kooperberg et al. 2001; Kooperberg and Ruczinski 2005), as well as data mining methods such as multifactor dimensionality reduction (MDR) (Hahn et al. 2003; Ritchie et al. 2001), tree-based methods such as classification and regression trees (CART) (Breiman et al. 1984) and Random Forests/Random Jungle (Breiman 2001; Schwarz et al. 2010), evolutionary algorithms designed for application to genetic data are grammatical evolution neural networks (GENN) (Turner et al. 2010a) and genetic programming neural networks (GPNN) (Motsinger et al. 2006b). The use of a wide variety of methods designed for gene–gene interaction analysis in pharmacogenomics studies is reviewed by Motsinger et al. (2007). For data integration methods, there are a number of methods emerging and more being developed all the time. For example, Huang et al. (2007) have been exploring the three-stage triangle method, where one investigates the association of SNPs with the trait to filter the SNP list, next these SNPs are tested for association with gene expression, and finally, those significant results are tested for association with the trait. Other approaches include using pathway analysis (Emilsson et al. 2008), Bayesian networks (Fridley et al. 2012), canonical correlation analysis (Chalise et al. 2012), and neural networks (Turner et al. 2010b; Holzinger and Ritchie 2012). We would direct readers to these manuscripts for a more in depth discussion of the different analytic methods appropriate for pharmacogenomics (Grady and Ritchie 2011; Motsinger et al. 2007; Holzinger and Ritchie 2012; Yan 2008).

## Success stories of pharmacogenomics: translational pharmacogenetics

As shown in Table 1, pharmacogenomics studies have observed a number of great successes in recent years. An entire area of pharmacogenomics that was not highlighted in the current review is cancer pharmacogenomics. The difference in cancer is that both germline DNA variation and somatic mutations in the tumor are part of the investigation. This changes some of the study design considerations described above, thus cancer is not a major focus of this review. However, it is important to note that many of the successes in translation of pharmacogenomics to the clinic are in the treatment of cancer such as *EGFR* tyrosine kinase inhibitors (TKIs) in the treatment of lung cancer (Yi et al. 2009) and *HER2*-directed therapies in the treatment of *HER2*-positive early-stage breast cancer (Arteaga et al. 2012). The following sections will highlight some of the pharmacogenomics results that have translated into precision medicine. All of these particular associations have been replicated in multiple studies and are also evaluated by the Clinical Pharmacogenetics Implementation Consortium (CPIC) of the Pharmacogenomics Research Network (PGRN) (Relling and Klein 2011). CPIC was established in 2009 to address the need for interpretation of genetic association results and guidance for clinicians so that pharmacogenetic tests could be used wisely in clinical care (Relling and Klein 2011). CPIC comprises physicians and researchers who are experts in pharmacogenetics, pharmacogenomics, and laboratory medicine many of whom are from the Pharmacogenomics Research Network (PGRN) and PharmGKB. CPIC has established a framework for evaluating levels of evidence needed to justify the implementation of a pharmacogenetic finding into clinical care. Their systematic approach is described in Relling and Klein (2011). In this review, we describe several success stories in pharmacogenetics with reported associations, CPIC evaluation, and clinical implementation (Relling and Klein 2011).

### Warfarin

Warfarin is often considered the poster-child for pharmacogenomics research. Warfarin is a widely used anticoagulant that needs to be closely monitored as patients whose warfarin levels are not maintained within its very narrow therapeutic index are at risk for clotting or bleeding. Common genetic variants in two genes, *CYP2C9* and *VKORC1*, have been associated with dosing variability along with several non-genetic factors, which when combined, explain up to 50 % of the variability in dose (Johnson et al. 2011). To be more specific in European descent individuals, *CYP2C9* and *VKORC1* explain up to 18 and 30 % of the variability, respectively; however, in individuals of other ancestry groups, the variants identified in Europeans explain much less of the variability. Routine clinical care in warfarin dosing is empirical; a physician prescribes a dose and monitors the patient closely to prevent under or over-anticoagulation (Johnson et al. 2011). Much research is ongoing to implement genetic testing into routine clinical care to use genotype to guide prediction of dose, including several genetic tests that are FDA approved (Johnson et al. 2011).

### Abacavir

Abacavir is a nucleoside reverse transcriptase inhibitor used in combination with other anti-retroviral medications indicated for the treatment of HIV. Abacavir is generally well-tolerated by patients, however, in 5–8 % of individuals undergoing treatment, a hypersensitivity reaction occurs (Martin et al. 2012). Symptoms of hypersensitivity include fever, rash, fatigue, cough, gastrointestinal symptoms, and dyspnea (shortness of breath). A genetic variant in HLA-B, HLA-B*57:01, is associated with this hypersensitivity reaction in about 6 % of patients (Martin et al. 2012). This association has been reviewed by Martin et al. (2012). Not only was this association observed in retrospective analyses of clinical trials, there was also a prospective, double-blind, randomized clinical trial of a genetic test to reduce adverse events through screening for HLA-B*57:01 before treatment (PREDICT-1) (Mallal et al. 2008). Based on the results of this trial and other supporting evidence, the FDA has implemented a black box warning in 2008 recommending that all patients be screened for HLA-B*57:01 before abacavir treatment. Abacavir is one of the best examples of implementation of pharmacogenomics into routine clinical care (Martin et al. 2012).

### Thiopurines

Thiopurines are a class of drugs used for nonmalignant immunologic conditions (specifically mercaptopurine and azathioprine), including inflammatory bowel disease, rheumatoid arthritis, and others, as well as lymphoid malignancies (mercaptopurine) and myeloid leukemias (thioguanine) (Relling et al. 2011). There is substantial evidence showing the association between TPMT genotype, thiopurine methyltransferase, and phenotypic variability in treatment response (Relling et al. 2011). Dosing recommendations have been developed and implemented, in the absence of a randomized clinical trial; however, a reduction in thiopurine-induced adverse events has been reported (Relling et al. 2011).

### Codeine

Codeine is an opioid analgesic used for the treatment of mild to moderately severe pain. Codeine is metabolized to morphine by *CYP2D6*, which has genetic variants that leave some individuals poor metabolizers and others ultrarapid metabolizers. More than 80 *CYP2D6* alleles have been identified by the Cytochrome P450 Nomenclature Committee (http://www.cypalleles.ki.se) and clinical phenotypes are known for some of these, but certainly not all of them. As shown by Crews et al. (2012), a *CYP2D6* score is calculated based on their genotypes at multiple alleles in the gene. A number of side effects have been reported for codeine use including gastrointestinal symptoms, drowsiness, dizziness, vomiting, sweating and several others (Crews et al. 2012). Case reports have reported severe and even life-threatening events in ultrarapid metabolizers (Crews et al. 2012). Genetic testing is available for *CYP2D6* in clinical care, although not performed by all physicians prescribing codeine. Based on the evidence, CPIC reports that alternative treatments be explored for patients who based on genetic testing are either poor metabolizers or ultrarapid metabolizers (Crews et al. 2012).

## Future directions in pharmacogenomics

Similar to all complex trait research in human genetics, pharmacogenomics is experiencing an explosion of data. The vast amount of data is extremely exciting for researchers, but brings with it significant challenges. Fortunately, as discussed previously, pharmacogenomics has a number of success stories to motivate and inspire future research endeavors. However, it is important to recognize that even for the traits with identified effects there is likely to be additional heritability that can be explained (Maher 2008). While estimating this heritability in PGx precisely is challenging (as family studies are not always readily available), the proposed heritability of most PGx traits exceeds that which has been explained so far. Thus, considering alternatives to the common variant hypothesis are warranted. Much like other traits, this additional heritability will be explored in:

- Rare variants, generated by genome sequencing experiments
- Combinations of common and rare genetic variants in polygenic and or predictive models
- Network and pathway analyses, including common and/or rare variants
- mRNA gene expression integrated with DNA sequence variation
- Gene–drug–environment interactions, including additional drugs and other environmental exposures

Fortunately, the barriers to data sharing are being reduced all the time, which makes it possible to assemble datasets with sufficient sample size to begin to consider effects like those listed above. The future of personalized medicine will likely involve predictive models composed of multiple variants and perhaps gene expression and environmental factors as well. We will learn the true complexity of pharmacogenomic traits.

Still, several success stories have been reported (described above) where pharmacogenomics discoveries have been made and many of these translated into the clinic. This process involves a significant amount of work, and the process has been slow for even the successful gene–drug relationships. This is due, in part, to a lack of specific guidelines on how to adjust medications on the basis of genetic testing results (Relling and Klein 2011). It is the goal of the CPIC (Relling and Klein 2011) to provide these guidelines to clinicians and laboratories. Important considerations that go into these guidelines include the results of pharmacogenomics studies, US Food and Drug Administration (FDA) label changes, and commercial sources release information for certain drugs (Relling and Klein 2011). It is certainly the case that personalized medicine, or precision medicine, is emerging in clinics around the world. However, best practices for making these translations are still in progress.

## Summary

Pharmacogenomics continues to expand in popularity as more genetic associations are uncovered. The nature of the effects in pharmacogenomics has been predominantly stronger and more interpretable than common disease associations. This is partially due to the known mechanism of action, metabolism, and transport for many pharmaceuticals. Another reason, and perhaps the more important one, is that pharmacogenomics can be translated into patient care in a near immediate course of action. For example, if a polymorphism is identified to be associated with drug dosing, physicians can change clinical care using genotype in the dosing algorithm. Likewise, if a polymorphism is associated with a serious adverse event, an alternative treatment could be selected for such patients. Finally, if it is known that treatment efficacy is optimal for one certain genotype group, while a similar drug is most efficacious for another genotype group, therapy can be personalized to achieve maximal success in patient care.
